# Proceedings of the Association for the Study of Comparative Psychology,in Connection with the Faculty of Comparative Medicine and Veterinary Science, McGill University, Montreal, for the Year 1889-90

**Published:** 1891-01

**Authors:** 


### SOCIETY PROCEEDINGS.

*Proceedings of the Association for the Study of Comparative Psychology, in connection with the Faculty of Comparative Medicine and Veterinary Science, McGill University, Montreal, for the Year 1889–90.*—This society met for the election of officers for the year, with the following results: Hon. President, Principal McEachran; President, Prof. Wesley Mills; First Vice-President, W. D. Smith, V.S.; Second Vice-President, Mr. Joel; Secretary-Treasurer, Mr. McGlua; Corresponding Secretary, Mr. Willyoung.

At a meeting held subsequently, the president in the chair, a large number of new members were proposed. The report of the retiring secretary-treasurer showed that the society had no debts, and that already a small amount had been expended in providing a nucleus for a library for the department of Comparative Psychology.

The inaugural address of the year was delivered by Prof. Charles McEachran. It was characterized by grace of style and excellence in manner of delivery, and was calculated to instruct, suggest and inspire. After alluding in general terms to the nature of the profession of veterinary medicine, he proceeded to show that a knowledge of the disposition and intelligence of animals was essential for the most successful treatment of these creatures when afflicted with disease. No one could become acquainted with the real nature of these animals without realizing that it was impossible to explain their mental manifestations and behavior by "instinct" alone. This position was supported by incidents that had either come under his own observation or that of trustworthy friends. Several groups of animals furnished these illustrations. One is so remarkable that it must be briefly retold. A gentleman in this city had two valuable pointers that were occasionally allowed the run of a back garden surrounded by a wall. One day, when thus enjoying themselves, the dogs entered the kitchen, and manifested unusual uneasiness, and then ran out, shortly to return still more anxious to communicate, evidently, some important discovery. They whined and tried to coax the servants to follow, one of the dogs actually pulling at the apron of the maid. The servants were constrained to accompany the dogs, to find, to their astonishment, that the source of the excitement was a living infant that some one had left just inside the garden wall, well wrapped up. What but a complicated mental state allied to our own could explain the behavior of these dogs? The address altogether was a charming and instructive one.

The Honorary President, Prof. D. McEachran, then made some remarks tending to reinforce the position of the speaker who had preceded him. Among other instances of general intelligence and sympathy with our own state of mind, he alluded to the behavior of a horse he rode when on his prairie ranch last summer. This animal, naturally wild, easily frightened and high-spirited, on one occasion stumbled, and, falling on his rider's leg, had disabled it. The horse, on rising, ran off to the distance of a quarter of a mile. Principal McEachran, hobbling toward a stream not far distant, cut a stick to act as support and protect himself against the wild cattle. In a little time the horse, of his own accord, approached, allowed himself to be quietly mounted and to be ridden equally quietly homeward. Such an unexpected realization that something was wrong, and such a corresponding change in his whole demeanor, called for explanation. Unless that animals reasoned and drew conclusions by some mental process closely allied to that employed by human beings, it was not possible to explain their conduct. In this view the president also concurred, and after he had spoken of the bright future he saw before the veterinary profession, and of the improvement that must come in the lot of the domestic animals when better understood, the society dispersed, to meet in about three weeks.

---

A meeting of the society was held, with the President, Prof. Wesley Mills, in the chair. The attendance was large. The Secretary reported the interchange of publications with the Toronto Humane Society. Several new members were elected.

The first paper of the evening was by Mr. Darling, on the psychology of a dog, a cross between a mastiff and a St. Bernard. The dog seemed to display the qualities of both of these breeds, and his general intelligence was high. His disposition was good, though, as usual, it was not improved by confinement on the chain. The essayist illustrated how, in various ways, this dog, like ourselves, profited by experience. Some of these instances were striking. The dog was averse to entering the water at command, and could never be induced to swim, but, nevertheless, amused himself by fishing. He stood in shallow water, and caught the fish by a snapdash at first, but later rushed at them before they were so near, and thus missed many. The fish were chewed up, but not eaten.

The next paper was by Mr. Willyoung. The writer endeavored to establish certain principles in regard to the lower animals, distinguishing between instinct and reason, and illustrating the resemblance between the mental processes of man and those of some of our domestic animals as well as of some wild ones. The case of a crow taken when young from the nest and tamed was surprising and interesting in the extreme. The bird's tongue had been operated on in some way, but exactly how was unknown. It learned to talk like a parrot, and displayed the feelings of jealousy, revenge, humor, etc., etc., in a way that was unmistakable. It was accustomed to express itself in both German and English. It had heard both these languages spoken in the family. This crow was accustomed to call the cows regularly and successfully at 5 o'clock.

Other animals were made use of to illustrate the writer's views.

A discussion was begun on these papers, but had to be postponed till the next meeting.

---

A meeting of the society was held, with the president in the chair. The attendance was good, and new members were elected. It was reported that several new works had been added to the society's library, and that others would be at once ordered from England.

Mr. Scott read a paper on animal intelligence, illustrating his views by such animals as the horse, the ox, the cat and the dog. Special attention was called to the power of discrimination, the retentiveness of memory and the appreciation of kind treatment shown by the horse. A horse has been known to recognize a man who had treated him harshly after years of separation. The writer of the paper referred to a certain horse that could distinguish between the sound of a locomotive whistle and that which indicated the noon hour and 6 o'clock, though they were very similar. This horse also objected to working over-time. Mr. Scott thought that the bovine species, especially bulls, possessed more intelligence than they usually got credit for.

The question of the intelligence of dogs, as evidenced in their cunning behavior when engaged in sheep worrying, and before and after it, led to considerable discussion. The general conclusion was to the effect that the dogs that usually worried sheep were not thoroughbreds of any variety, but mongrel dogs—canine tramps, in fact.

The president instanced the case of a dog cruelly left behind when the family moved away, that, to support itself, paid regular visits to the hen-roosts of the vicinity, and helped himself to the fowls.

Fowls supposed to be killed by foxes were sometimes, in reality, killed by these prowling dogs. Such dogs should be destroyed. They brought the whole canine race into disrepute with the undiscerning. Taxing dogs was but a crude way of regulating the dog nuisance. Thoroughbred dogs should, for many reasons, be exempt, while vagrant curs should be destroyed.

The whole canine race should no more be held responsible for the doings of its worst members than the whole human family for the behavior of the criminal classes. For both alike, civilization was responsible. The dog, above all, is what man makes him. The individual that breeds and trains high-class dogs (or animals of any kind) is doubly a benefactor. He renders the excuse for keeping curs less valid, and he furnishes what is worth having. It was the duty and the privilege of the society to diffuse such views.

Discussion on the paper of the previous meeting was resumed.

One of the members related a remarkable instance of intelligence in a mule. This animal had been employed to draw a car loaded with coal from the mine to the dump. He was usually unhitched there, and being replaced in position drew back the empty car. On one occasion he had accidentally got free, and after taking a drink from the river near by returned and pushed the car before him with his breast. After this he was used regularly for work in this way.

The President finally gave some hints as to the anatomy and physiology of the nervous system, which he thought would assist in making the whole subject of animal intelligence more scientific and comprehensive.

---

A meeting of the Society for the Study of Comparative Psychology was held in the lecture room of the Faculty of Comparative Medicine and Veterinary Science, the President, Prof. Wesley Mills, in the chair.

The executive reported that a new form of diploma would be prepared to suit the change in the relations of the school to the university of which it now was a faculty.

The first paper of the evening was read by Mr. Crossman, entitled "Instinct and Reason." The writer thought that no one who studied the development of an intelligent dog from puppyhood to maturity could believe that he was guided only by instinct. "If all the philosophers in the world should try to convince me, I should never be able to persuade myself that an animal is a mere machine." The position taken was illustrated by instances that came under the writer's own observation, especially in the horse. He instanced the case of a mare that used to let down bars in order to get into an adjoining field. This required more than instinct, and it could not have been by instinct that her colt learned the same thing from its mother, for instinct implies action independent of experience or comparison. The mother did not soon forget the habit, but proceeded to let down the bars at once after a year's interval. The paper was evidently the result of independent observation and thinking.

The next paper, by Mr. Walsh, was on a similar subject, but the handling was somewhat different. This writer discussed incidentally the senses in our domestic animals, in races of uncivilized men and in the man of the most advanced civilization, showing that in the latter certain senses, as smell, have undergone a partial impairment from disease, owing to the necessity for them being less urgent. Mr. Walsh proceeded to show how unfavorable in most cases circumstances were for the development of the higher mental aud moral qualities of animals, owing to man's neglect or positive ill-usage. It was of no small importance to breed from animals that showed the best disposition and the highest intelligence.

The influence of one animal over its companions was then illustrated by several forcible examples, showing that in this respect they were wonderfully like ourselves. Both the papers were calculated to produce a better feeling toward our dumb companions and a more moderate estimate of our own powers.

The discussion was so free that the evening did not suffice to finish it.

Several members illustrated the intelligence of animals by their ability to let down fences, notwithstanding many mechanical contrivances, and it was seen that the habits of animals were, like our own, capable of being very strongly fixed. One of the members related the case of a Collie dog that used to open a door to enter the house. He was told on one occasion to go back and shut the door. This he did, and continued to do so of his own accord always afterward. It was pointed out that certain acts in the lower animals were the result of instinct primarily, but as modified by intelligence; in fact, that there is much in the mental life of man of the same character. The president referred to the interest he found attaching to the study of the different dogs in his own kennel, and especially in noting the individuality of each, and, at the same time, the modification of each one's character by the other. An ill-behaved dog would spoil to a degree a whole kennel, just as a bad boy in a school may lower the tone of a whole class. And in every kennel the strong characters among the dogs made themselves felt even in puppyhood. He was accustomed to have his dogs' sense of right and justice cultivated. No dog was ever allowed, under any circumstances, to take another's food, his bed or even his bone. The dogs soon learned to respect each other's rights independent largely of might.

---

The next meeting of the society was held in the usual place, the president in the chair.

Mr. Scanlan read a paper, in which he maintained that the behavior of our domestic animals could not be explained by instinct alone, but that their actions showed that with instinct we must recognize that intelligent adaptation which implies the power to draw inferences and profit by experience. These animals seemed also to experiences feelings closely akin to our own. The writer advanced facts that had come under his observation in the case of the horse, the dog, the cow and the cat. These recitals were all pertinent and convincing. The case of a dog that feigned sleep after stealing from the kitchen, and so successfully as to escape detection until watched and caught in the act, was clear evidence that instinct alone could not explain the action of animals. The writer stated that he had a friend who, when studying at the Conservatory of Music, observed that a certain spider always left its web and descended to near where he was when playing the violin. Two cats that at first opened the door accidentally, when attempting it together afterward acted regularly in concert to accomplish this purpose.

The president here stated that he had an Irish setter which, when a puppy of seven months, would open nearly all kinds of doors when anxious to get out to follow older dogs, and used a great many devices to effect this.

A paper was read by Mr. Hayman, entitled "Our Dumb Companions; their Intellect and Treatment." He contended that the best way to advance the interests of dumb animals was to make their nature understood. In conclusion, he said he had always found that kindness, patience and good temper had their reward in dealing with both man and the lower animals.

The discussion was animated, and had to be postponed for continuation at the next meeting.

The president asked those who had had most experience with balky horses to state their views, with reasons, on the question: Are balky horses less or more intelligent or sensitive than others?

This subject was discussed with much point and clearness. The general impression was that there are different kinds of balky horses, some very stupid and stubborn, others more than usually intelligent, and others that were highly nervous and easily discouraged. All seemed to agree that much depended on the driver and the treatment the animals received.

The president suggested that it would be greatly in the interests of the horses themselves, and also the public that this subject should be dealt with in a paper and be fully discussed before the society at some future meeting.

---

The last regular meeting of the Society for the Study of Comparative Psychology was held in the lecture room of the Faculty of Comparative Medicine and Veterinary Science, with the President, Prof. Wesley Mills, in the chair and a large attendance of members.

The first paper of the evening, by Mr. McGue, the case of a Gordon setter dog, elicited some discussion and gave rise to many questions. This dog belonged to a gatekeeper on a line of railway. He was accustomed to wake his master, who was required to be on duty early every morning. The animal would lie with his head on the rails, and seemed to be able to recognize the approach of a train at a great distance; and this was the more singular as he was slightly deaf, and had for that reason been discarded as a hunting dog. He also had learned to know the trains that stopped from those that did not before they came into view, and behaved accordingly.

Mr. Barton read a paper entitled "Reasoning Power in Animals." The paper showed that various authors had been studied and carefully compared by the writer, and though somewhat lacking in originality, it embodied a good discussion of the subject. The distinction claimed for man, that he alone is capable of true reflection and of self-consciousness was considered, also Max Muller's dictum that thinking is only possible through language, and that, therefore, man alone could be said to think.

The president pointed out that Max Muller's position was a begging of the question. No doubt man was capable of a kind of thought of which no other animal could give evidence. At the same time it was to be borne in mind that the greater part of man's thinking was not in the abstract. He believed that for the mass of men a large part of their thinking, if such we are to call it, was in images, without the use of languages at all, and by rapid inference, in which we resemble our dumb companions, though in rapidity of inference they often excel us, as is admitted by Max Muller himself.

It was likely, he said, the Gordon setter, being rather deaf, availed himself of the vibration of the earth and the rails to learn by contact of the approach of trains. By the law of association these two things had become fixed in the dog's organism, and were revived simultaneously in his consciousness. In such respects the nature of the lower animals and man do not differ essentially. All animals think within the limits of their experience.

After some further discussion and hints from the president as to summer study, the society adjourned.

